# Longitudinal Assessment of Lung Cancer Progression in Mice Using the Sodium Iodide Symporter Reporter Gene and SPECT/CT Imaging

**DOI:** 10.1371/journal.pone.0169107

**Published:** 2016-12-30

**Authors:** Dominique N. Price, Amber A. McBride, Martina Anton, Donna F. Kusewitt, Jeffrey P. Norenberg, Debra A. MacKenzie, Todd A. Thompson, Pavan Muttil

**Affiliations:** 1 Department of Pharmaceutical Sciences, College of Pharmacy, University of New Mexico Health Sciences Center, Albuquerque, New Mexico, United States of America; 2 Sandia National Laboratory, Albuquerque, New Mexico, United States of America; 3 Institute of Molecular Immunology/Experimental Oncology and Therapy Research, Klinikum rechts der Isar der Technischen Universität München, Munich, Germany; 4 Department of Pathology, University of New Mexico School of Medicine, Albuquerque, New Mexico, United States of America; 5 New Mexico Center for Isotopes in Medicine, Albuquerque, New Mexico, United States of America; 6 University of New Mexico Comprehensive Cancer Center, Albuquerque, New Mexico, United States of America; Wayne State University, UNITED STATES

## Abstract

Lung cancer has the highest mortality rate of any tissue-specific cancer in both men and women. Research continues to investigate novel drugs and therapies to mitigate poor treatment efficacy, but the lack of a good descriptive lung cancer animal model for preclinical drug evaluation remains an obstacle. Here we describe the development of an orthotopic lung cancer animal model which utilizes the human sodium iodide symporter gene (hNIS; SLC5A5) as an imaging reporter gene for the purpose of non-invasive, longitudinal tumor quantification. hNIS is a glycoprotein that naturally transports iodide (I^-^) into thyroid cells and has the ability to symport the radiotracer ^99m^Tc-pertechnetate (^99m^TcO_4_^-^). A549 lung adenocarcinoma cells were genetically modified with plasmid or lentiviral vectors to express hNIS. Modified cells were implanted into athymic nude mice to develop two tumor models: a subcutaneous and an orthotopic xenograft tumor model. Tumor progression was longitudinally imaged using SPECT/CT and quantified by SPECT voxel analysis. hNIS expression in lung tumors was analyzed by quantitative real-time PCR. Additionally, hematoxylin and eosin staining and visual inspection of pulmonary tumors was performed. We observed that lentiviral transduction provided enhanced and stable hNIS expression in A549 cells. Furthermore, ^99m^TcO_4_^-^ uptake and accumulation was observed within lung tumors allowing for imaging and quantification of tumor mass at two-time points. This study illustrates the development of an orthotopic lung cancer model that can be longitudinally imaged throughout the experimental timeline thus avoiding inter-animal variability and leading to a reduction in total animal numbers. Furthermore, our orthotopic lung cancer animal model is clinically relevant and the genetic modification of cells for SPECT/CT imaging can be translated to other tissue-specific tumor animal models.

## Introduction

Lung cancer remains the leading cause of cancer deaths among both men and women with a five-year survival rate of 18.7% [1]. Research in the areas of lung cancer prevention, treatment, and diagnostics continues to make progress, with the discovery of novel immune check-point inhibitors and chemotherapies among the successes of the last decades.

Animal tumor models are critical for the development of novel cancer chemotherapeutics. However, current lung cancer animal models cannot quantify tumor burden longitudinally throughout treatment precisely and requires the sacrifice of animals at several time points throughout treatment. This results in studies with large animal cohorts to quantify tumor burden at multiple time points throughout treatment, leading to variability in tumor sizes because of inter-animal differences. Furthermore, many of these animal models do not closely resemble the clinical features of the human cancer-type being studied [2,3]. The use of *in vivo* imaging modalities, like computed tomography and optical imaging, have made it possible to study tumor growth or treatment longitudinally in a single animal; however precise tumor imaging has been limited by problems with imaging sensitivity, spatial resolution, and the ability to precisely quantify tumor burden and growth [4].

Genes encoding the intracellular transport, binding, or uptake of radioactive tracers in place of normal proteins have emerged as an innovative strategy for non-invasive visualization of tumor growth in animal models [5–7]. Reporter gene imaging is based on vector-mediated overexpression of a transgene that is not normally expressed in the host cells [8,9]. Furthermore, the feasibility of non-invasive imaging using a radiolabeled reporter probe and single-photon emission computed tomography (SPECT) or positron emission tomography (PET) has been successfully demonstrated in animals [10–12].

The human sodium iodide symporter (hNIS) is an integral plasma membrane glycoprotein that mediates active iodine (I^-^) uptake in tissues such as the thyroid, salivary glands, gastric mucosa, and lactating mammary glands [13]. hNIS-mediated I^-^ uptake is an active transport process that occurs against the electrochemical gradient and uses the sodium gradient generated by the Na^+/^K^-^ ATPase to co-transport two Na^+^ and one I^-^ ion across the basolateral membrane of cells [14,15]. Radioiodine and ^99m^Tc-pertechnetate (^99m^TcO_4_-) are two well-established radiotracers for the hNIS gene and are regularly used for diagnostic scintigraphic imaging of the thyroid [15,16]. Both radiotracers are widely available without complicated labeling procedures, and imaging can be performed with a conventional gamma camera [17,18]. Recent work has exploited the ability of hNIS to accumulate a radiotracer endogenously, and shown its utility by transfecting /transducing cells and expressing them in transplanted tissue for treatment and non-invasive imaging purposes [19–23].

In the present study, we show the development and optimization of a lung cancer mouse model that has longitudinal imaging capabilities using a hNIS-transduced system. Toward this goal, A549 lung adenocarcinoma cancer cells were modified to stably express the hNIS protein as an imaging reporter. We established a subcutaneous xenograft and an orthotopic xenograft tumor model in nude mice where tumor growth kinetics were quantified non-invasively at two time points in the same animals using a small animal SPECT/CT imager. This orthotopic lung cancer mouse model allows for sensitive assessment of cancer chemotherapeutics longitudinally. Furthermore, this work can be translated to other tissue-specific cancers and could inform further development of novel tissue-specific cancer imaging animal models.

## Materials and Methods

### Cell lines

The human epithelial adenocarcinoma cell line CCL-185^™^ A549 was purchased from American Type Culture Collection (ATCC, Manassas, VA). Unmodified A549 cells and genetically modified A549 cells were cultured in F-12K nutrient mixture (kaighn’s modification) 1x media from Invitrogen (Grand Island, NY), supplemented with 5% fetal bovine serum and 1% penicillin-streptomycin from Invitrogen (Grand Island, NY). Cells were maintained at 37°C under an atmosphere of 5% CO_2_.

### Plasmid vector transfection

The A549-hNIS cell lines were generated by selecting for stable transfectants after plasmid transfection. Primers were designed based on the cDNA sequence SLC5A5 (Entrez Nucleotide, National Center for Biotechnology Information, Bethesda, MD) and amplified from a human cDNA clone (ATCC 11047241) to form a 1,932 basepair insertion into the multiple cloning site (MCS) of the pIRES2DsRedExpress vector (Clontech, Mountain View, CA). The IRES2DsRedExpress vector is a bicistronic expression vector that is driven by a constitutively active human cytomegalovirus promoter located upstream of the MCS and includes a neomycin-resistance cassette to allow for G418 selection. The modified vector was transfected into A549 cells using the FuGene 6 reagent (Promega, Madison, WI) according to the manufacturer’s protocol. Modified clones were plated in serial dilution and selected using 500 μg/ml of G418 (VWR, Radnor, PA, USA) for 2 weeks. Transiently transfected cells were also generated using the FuGene 6 reagent. A549-pDNA clones were screened for ^99m^TcO^4-^ uptake activity and cells with the highest uptake were chosen for subsequent *in vivo* studies.

### Lentiviral vector transduction

The A549-hNIS cell line was also generated using a lentiviral vector that encodes the hNIS reporter gene or eGFP driven by a phospho-glycerate kinase housekeeping gene (PGK) promoter or spleen focus forming strong viral (SFFV) promoter. Lentiviruses were produced in 293T cells according to standard protocols; A549 cells were then infected with the virus-containing medium in the presence of 8 μg/ml polybrene. After infection, fresh F-12K medium was added to allow cells to recover for 24 to 48 h. Clones were assessed for surface hNIS expression using a monoclonal antibody against the extracellular domain of hNIS (VJ2; a gift S. Costagliola, Free University Brussels, Belgium to the Institute of Experimental Oncology and Therapy Research, Klinikum rechts der Isar der Technischen Universität München) and an anti-mouse FITC-labeled monoclonal antibody (STAR 70, Bio-Rad AbD Serotec GmbH, Puchheim, Germany), and analyzed by flow cytometry using a FACS Canto (BD Biosciences, Heidelberg, Germany) and the CellQuest software (BD Biosciences) as performed previously [24].

### hNIS—mediated ^99m^TcO_4_- uptake assay

Modified A549 cells were screened for stable expression of hNIS based on ^99m^TcO_4_^-^ uptake activity. Cells were plated 24 hours before the start of the assay at a density of 1 x 10^6^/well in a 12 well plate. One hour prior to the start of the ^99m^TcO_4_^-^ uptake assay, cells were counted by hemocytometer using a 0.4% solution of trypan blue (HyClone^™^ Trypan Blue Stain, GE Healthcare Life Sciences, Logan, Utah, USA). Cells were treated with 37 kiloBecquerel (KBq, equivalent to 1.0 microcurie) of ^99m^TcO_4_^-^ (Cardinal Health, Albuquerque, NM) for 1 hour at 37°C with 5% CO_2_. Cells were washed twice with cold PBS. One mL of 20mM sodium acetate (Sigma-Aldrich, St. Louis, MO) in PBS was added to each well, incubated at room temperature for ten minutes and supernatant removed. One mL of 0.1 M NaOH (Sigma-Aldrich, St. Louis, MO) was added to each well to de-adhere and lyse cells. The cell lysate was collected and radioactivity counted by a gamma counter (Wallac Wizard 1470–005, Perkin Elmer). For radiotracer uptake in these cells, ^99m^TcO_4_^-^ activity (counts per minute) for each sample set was divided by the total cells in each well to normalize the data. Those cells with the highest ^99m^TcO_4_^-^ activity were chosen for subsequent *in vivo* studies.

### Animal care

Male, 6-week old, HSD:Athymic Nude-*Foxn1*^*nu*^ mice (Harlan Sprague Dawley, Inc., Indianapolis, IN, USA) were housed and maintained in specific pathogen-free conditions in a facility approved by the American Association for Accreditation of Laboratory Animal Care under National Institutes of Health Guidelines. Food and water were provided *ad libitum* to the animals in standard cages. All experiments were performed in accordance with the guidelines of the Institutional Animal Care and Use Committee at the University of New Mexico under the approved IACUC protocol number HSC100853. Animals were monitored for morbidity or signs of distress throughout the study. Humane endpoints for these studies were based on weight loss (>10–15%) and clinical observations for behavior (decreased activity, hunched posture, shivering, labored breathering, moribund). We did not observe any adverse event during our studies, including signs of illness or mortality prior to experimental endpoint.

### Development of subcutaneous xenograft tumors

Plasmid or lentiviral vector-carrying A549-hNIS cells (3 x 10^6^ cells in 100 μl per injection site) were suspended in 1:1 F 12K media and matrigel (BD Biosciences, San Jose, CA, USA). Mice were anesthetized by continuous flow of 3% isofluorane (2.5% LPM oxygen), and A549 cells were injected subcutaneously (s.c.; 25 gauge, 5/8” needle). Animals were observed for 45 to 60 minutes after tumor inoculation, until fully recovered. Each animal received 4 tumor inoculations; one each in the: a) left anterior lateral thoracic wall; b) right anterior lateral thoracic wall; c) left posterior lateral aspect of the flank; d) right posterior lateral aspect of the flank [25]. Tumor growth was monitored with a digital caliper every other day. Tumor volume was estimated according to the following formula: V = (S^2^ x L)/2, in which S and L are the smaller and larger measures of the tumor, respectively [26].

### Development of orthotopic xenograft lung tumors

Plasmid or lentiviral vector-mediated A549-hNIS cells were prepared as described previously. Mice were anesthetized by continuous flow of 3% isofluorane (2.5% LPM oxygen) and a 5 mm skin incision was made to the left chest, ~ 5 mm below the sternum. A syringe with a 25 gauge hypodermic needle containing A549-hNIS cells was advanced to a depth of 5 mm in the mediastinum of the left lung between the 5^th^ and 6^th^ intercostal rib space (protocol modified from Ichite et. al. and Saha, et al.) [27,28]. 3 x 10^6^ cells in 100 μl were delivered per injection site.

### SPECT/CT imaging

Tumors (s.c. and orthotopically transplanted) were imaged in mice using SPECT and CT (NanoSPECT/CT, Mediso USA). Mice were anesthetized with 3% isofluorane (2.5% LPM oxygen) and 37 MBq (1.0 mCi) of ^99m^TcO_4-_ was administered by tail vein injection. One hour post ^99m^TcO_4_^-^ injection, SPECT/CT imaging of mice was performed. Mice were placed on a heated bed and remained anesthetized at 2% isofluorane (2.5% LPM oxygen) during the entire imaging process and were monitored to ensure proper recovery from the anesthesia.

### Analysis of SPECT/CT imaging

An automated library-based segmentation technique with a high-density lung tissue was used to segment the lungs from the CT image data, employing manual quality control and intervention as necessary. For the SPECT and CT data analysis, independently, the Otsu method was applied within segmented lung regions to calculate the tumor threshold [29]. Lung regions where intensity exceeded the threshold were classified as tumor voxels. The final tumor area margin was defined as the intersection of the modality-specific tumor regions and healthy tissues.

Both CT and the SPECT were resampled to an isotropic voxel size of 0.2 mm. The SPECT was registered to the CT manually using a linear interpolator. The CTOtsu and NMOtsu ROIs were generated using Otsu’s method using 300 histogram bins. VivoQuant 1.23 (inviCRO, LLC) was used to view and process the data. The topogram/scan range was between 133/140–232/233, 65 kVp, 500 ms. CT was acquired using 180 projections, 1.5 pitch, with an acquisition time of 3 minutes. SPECT was acquired using 32 projections with an acquisition time of 30 minutes. No smoothing was applied to the data.

### hNIS mRNA Expression

hNIS mRNA expression was determined using real-time quantitative reverse transcriptase polymerase chain reaction (qRT-PCR). Tissue was flash frozen in liquid nitrogen and stored at -80°C. Total RNA was extracted using Trizol reagent (Invitrogen) and cDNA was prepared using the High Capacity cDNA Reverse Transcription Kit (Applied Biosystems, Foster City, CA). qRT-PCR was performed using an Applied Biosystems 7900HT Fast Real-Time PCR System (Carlsbad, CA). The cycling parameters were 95°C for 10 min followed by 40 cycles of 95°C for 15 sec, and 60°C for 1 min. Forward and reverse hNIS primers and the normalization control gene glyceraldehyde 3-phosphate dehydrogenase (GAPDH) were purchased from Integrated DNA Technologies (Coralville, IA). The hNIS forward primer sequence was 5′-CAGTGGCCCCCAAGGAAGAAGT-3′ and the reverse primer sequence was 5′-GGAAGCCAGGGGGCTTCTTGT-3′. hNIS mRNA expression levels normalized to GAPDH were determined using the ΔΔCt method.

### Tumor histology

Inflated lungs and trachea were removed *en bloc*. Caution was taken during fixation to prevent lung collapse, deflation, tissue structure disruption, and fixation artifacts that could lead to alveolar wall thickening, hypercellularity, change in shape or volume, and blood in the lungs [30]. Briefly, a cannula was inserted into the trachea and fixed with a ligature. The lungs were fixed by infusion of fixative (10% formalin) through the cannula by continuous release of fixative under pressure for 15 minutes [31]. Tissues were stored in 10% formalin until processing and then embedded in paraffin. Seven-micron sections were sliced in a sagittal plane and stained with hematoxylin and eosin (H&E). Tumor samples were analyzed for histological changes including the presence of tumor necrosis, apoptosis, mitotic activity, and cytologic atypia.

### Statistical analysis

All statistical analyses were performed using Graphpad Prism statistical software (GraphPad Software, San Diego, CA). A one-way ANOVA with Tukey’s multiple comparison post-test was used for parametric data and a one-way ANOVA Kruskal-Wallis with Dunn’s multiple comparison post-test was used for multiple comparisons of non-parametric data. Additionally, for two variable analysis, a two-way ANOVA with Tukey’s multiple comparison post-test was used for parametric data and a two-way ANOVA with Holm-Sidak’s multiple comparison post-test was used for non-parametric data. Statistical significance was reported as *, P < .05; **, P < .01; ***, P < .001; ****, P < .0001.

## Results

### hNIS expression in A549 cells

Two methods for hNIS expression in A549 cells were employed, plasmid transfection and lentiviral infection, and were evaluated for stable hNIS integration and *in vitro* ^99m^TcO_4_- uptake. The pIRES2-DsRed-Express plasmid was used to transfect A549 cells and G418 antibiotic was added to select for hNIS expression (abbreviated A549-pDNA hereafter). For lentiviral vector transduction of A549 cells, two different promoters were used to drive hNIS expression. Flow cytometric analysis showed 30.6% hNIS positive cells for the PGK promoter and 73.0% hNIS positive cells for the SFFV promoter. The SFFV promoter further resulted in a 2.9-fold stronger mean fluorescence intensity than the PGK promoter (S1 Fig), therefore the lentiviral vector cells containing the SFFV promoter to drive hNIS expression (abbreviated A549-LV hereafter) were used for *in vivo* studies.

**Fig 1:**
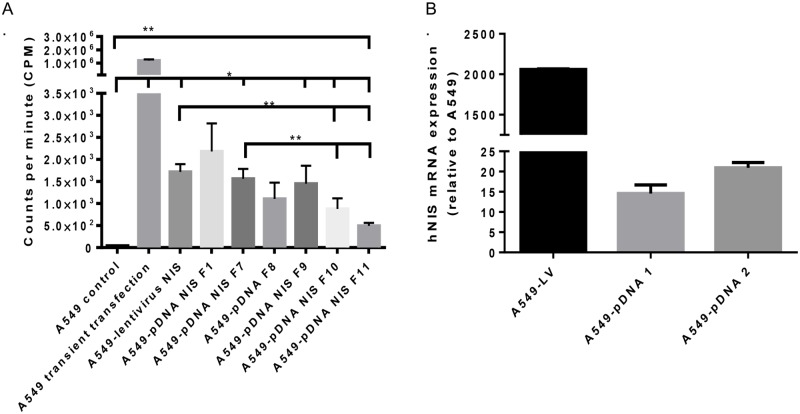
Characterization of lentiviral and plasmid vector-mediated hNIS expression in A549 lung cancer cells. A) pDNA vector-mediated and lentiviral vector-mediated cell lines were characterized by *in vitro* uptake of ^99m^TcO_4_^-^. B) Three cell lines were selected for *in vitro* mRNA hNIS expression analysis: A549-LV, A549-pDNA 1 and A549-pDNA 2. A one-way ANOVA with Tukey’s multiple comparison post-test and a Kruskal-Wallis ANOVA with Dunn’s multiple comparison test were used to determine statistical significance,** p < .01; data shown with standard error of the mean.

To validate the activity of hNIS in both transfected and transduced cells, an *in vitro* hNIS—mediated ^99m^TcO_4_- uptake assay was performed to characterize A549-pDNA and A549-LV cells. ^99m^TcO_4_- uptake analysis of transiently-transfected A549 cells (no incorporation of hNIS into the A549 cell genome) showed significantly higher ^99m^TcO_4_^-^ uptake compared to A549 vehicle (unmodified cells), and was used as positive control of hNIS expression (Fig 1A). Untransfected A549 cells showed no hNIS activity. A549-NIS transfected and transduced clones were compared for ^99m^TcO_4_^-^ uptake as evidence of stable hNIS incorporation. The A549-pDNA NIS F1, F9, and A549LV NIS had the highest ^99m^TcO_4_^-^ uptake activity at about 1.5 x 10^3^ to 2.2 x 10^3^ counts per minute (Fig 1A; 7 of 34 cell lines assessed including A549-lentivirus NIS, A549-pDNA NIS F1 and A549-pDNA NIS F9 named A549-LV, A549-pDNA 1 and A549-pDNA 2, respectively; these abbreviations are used going forward). These three cell lines were selected for their increased hNIS functionality and further used for *in vivo* studies.

Real-time quantitative PCR (qRT-PCR) was used to measure hNIS expression of A549-LV, A549-pDNA 1, and A549-pDNA 2 relative to A549 unmodified cells. A549-LV vector-mediated cells expressed 2060-fold more gene expression than A549 control cells. A549-pDNA 1 and A549-pDNA 2 cell lines expressed 14-fold and 20-fold more hNIS than A549 unmodified cells, respectively (Fig 1B). Based on qRT-PCR results, we expected the A549-LV cells would best sequester ^99m^TcO_4_^-^ in A549 cells in the tumor mouse models.

### In vivo SPECT/CT imaging of subcutaneous xenograft tumors

**Fig 2:**
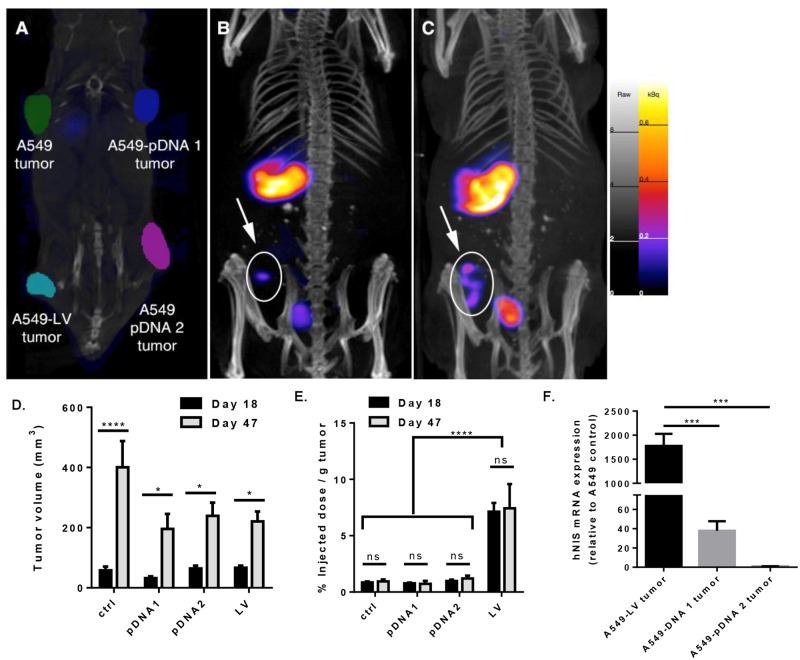
Subcutaneous xenograft tumor radioactivity and quantification of A549 control tumors, A549-lentiviral vector-hNIS modified tumors, and A549-pDNA vector-hNIS modified tumors. A) Orientation of xenograft tumors as shown by phantoms imaged by CT: upper left: A549 control tumor; lower left: A549-LV tumor; upper right: A549-pDNA 1 tumor; and upper right: A549-pDNA 2 tumor, B & C) Tumor volume quantification by SPECT/CT and ^99m^TcO_4_^-^ radioactivity on day 18 (**B**) (n = 6; representative images shown) and day 47 (**C**) (n = 7; representative images shown). Spleen and bladder are visible at day 18 and 47. D) Tumor growth comparison of A549 control tumors, A549-LV tumors, and A549-pDNA tumors as measured by volume (mm^3^). E) % injected dose ^99m^TcO_4_^-^ per gram tumor (%ID/g) after sacrifice. F) mRNA expression of NIS relative to A549 control. A one- and two-way ANOVA with Tukey’s multiple comparison post-test was used to determine statistical significance,** p < .01; ***p < .001; ****p < .0001; data shown with standard error of the mean.

hNIS expression in tumors was first evaluated in a xenograft mouse model. Tumors were allowed to grow for a total of 47 days and imaged by SPECT and CT at 18 and 47 days (Fig 2). Phantom images show subcutaneous (s.c.) xenograft tumor placement and overall tumor growth, qualitatively determined by CT imaging (Fig 2A). A preliminary ^99m^TcO_4_^-^ uptake study was performed to determine optimum radioactivity in tumors compared to background signal at 30 minutes, 1 hour, 3 hours, 6 hours and 24 hours post ^99m^TcO_4_^-^ injection. One-hour post-^99m^Tc injection was found to be optimal.

At day 18 after tumor inoculation, only the A549-LV tumor was detectable by non-invasive imaging (Fig 2B). Again, at day 47 after tumor inoculation, only the A549-LV tumor was observed (Fig 2C) even though A549 control, A549-LV, A549-pDNA 1, and A549-pDNA 2 tumors were visualized and quantified by CT and SPECT (Fig 2D). ^99m^TcO_4_^-^ uptake (% ID/g) was determined in the tumor tissue by SPECT voxel analysis and showed over 7-fold more radioactivity per gram of tumor in the A549-LV tumor compared to the A549 control tumor, A549-pDNA 1 and A549-pDNA 2 tumors even though all the tumor sizes were an average of 200 mm^3^ on day 47 after tumor inoculation (Fig 2E–2D). Additionally, it was important to note that radioactivity (% ID/g) remained constant in all tumors (per gram of tumor mass) from day 18 to 47, suggesting stable hNIS incorporation throughout tumor growth with both plasmid and lentiviral vectors.

### Quantification of hNIS expression in tumors

We further corroborated hNIS expression by conducting qRT-PCR of mRNA from excised tumors at day 47, to assess whether hNIS integration remained stable (Fig 2F). A549-pDNA tumors had low levels of hNIS mRNA. A549-LV tumors expressed an average of 46-fold more hNIS mRNA than A549-pDNA 1, and 2145-fold more hNIS than A549-pDNA 2 (Fig 2F).

### *Histopathological analysis* of primary tumors in the s.c. xenograft

**Fig 3:**
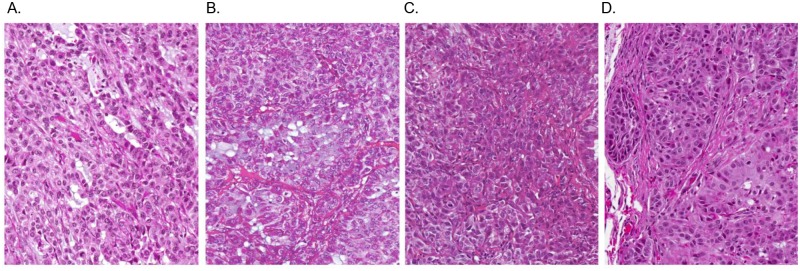
H&E staining of subcutaneous-induced xenograft tumors in athymic Nude-Fox1^nu^ mice. Tumors were induced by injecting A549-hNIS cells that were genetically modified with either plasmid or lentiviral vectors. Mice were sacrificed at 47 days post tumor cell inoculation. An H&E tumor section of the lung is shown resulting from: A) A549 control tumor; no genetic modification. B) A549-pDNA 1 tumor. C) A549-pDNA 2 tumor. D) A549-LV tumor. (n = 3; all sections shown at 20x magnification; representative images shown)

To confirm SPECT/CT detection of tumor burden in mice, we performed histological analysis of H&E stained tumor tissues. At sites of s.c. xenograft transplantation of A549-pDNA and A549-LV modified cells lines, masses composed of large neoplastic epithelial cells were found (Fig 3B–3D). Nests of round to polygonal neoplastic epithelial cells were incompletely separated by variable amounts of collagenous stroma. Tumor cells had abundant cytoplasm, relatively distinct cell borders, and centrally-located round to oval nuclei often containing a single prominent nucleolus. All tumors showed signs of necrosis. Larger tumors had variable necrosis, both in terms of extent and pattern. However, no metastasis was evident in mice with s.c. xenograft transplantation via visual examination.

### In vivo SPECT/CT imaging of orthotopically-induced xenograft tumors

**Fig 4:**
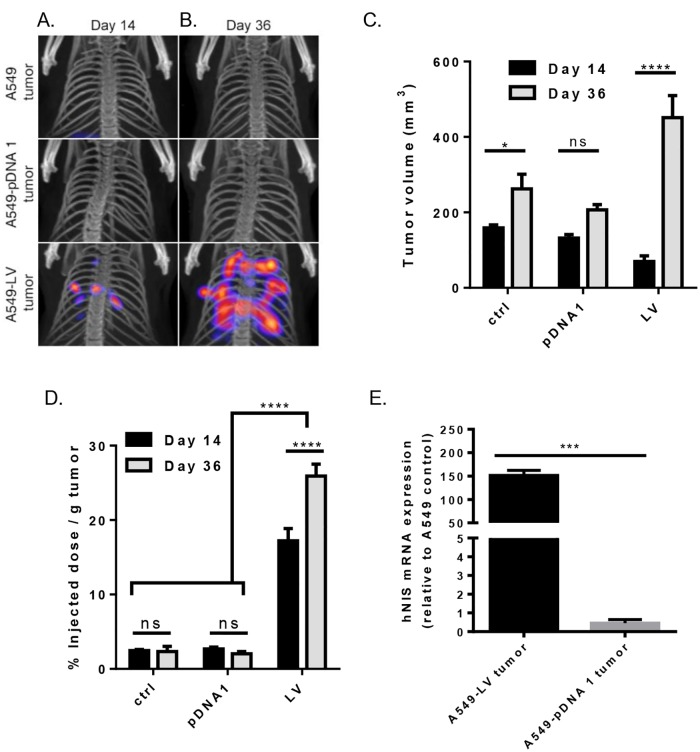
Tumor radioactivity and quantification of orthotopically-injected A549 control tumors, A549-lentiviral vector-hNIS modified tumors, and A549-pDNA vector-hNIS modified tumors. A & B) Tumor volume quantification by SPECT/CT and ^99m^TcO_4_^-^ radioactivity on day 14 (A; respective tumor groups shown horizontally; representative images shown) and day 36 (B; respective tumor groups shown horizontally; representative images shown). C) Tumor growth comparison of A549 control tumors, A549-LV tumors, and A549-pDNA tumors as measured by volume (mm^3^). D) % injected dose ^99m^TcO_4_^-^ per gram lung tumor (%ID/g) after sacrifice. E) mRNA expression of NIS relative to A549 control. A one-way ANOVA with Tukey’s multiple comparison post-test and a two-way ANOVA with Holm-Sidak’s multiple comparison test was used to determine statistical significance,** p < .01; ****p < .0001; data shown with standard error of the mean.

To further assess hNIS as an imaging tool for lung cancer, we examined its utility in an orthotropic xenograft mouse model. Like the s.c. xenograft tumor model, orthotopically-transplanted xenograft tumors were imaged by SPECT/CT at 2 time points (day 14 and 36 after tumor inoculation). SPECT images show radioactivity for A549-LV tumors at day 14 (Fig 4A). A significant increase in radioactivity was seen at day 36 for A549-LV tumors compared to day 14 (Fig 4B). However, no radioactivity was observed for the A549-pDNA tumor at either imaging time point even though CT detected similar tumor volume among all tumor transplanted groups (Fig 4A–4C). We observed an increase in ^99m^TcO_4_^-^ uptake (% ID/g) in all mice in the A549-LV tumor group from day 14 to 36, resulting in an average 1.5-fold increase in ^99m^TcO_4_^-^ uptake (% ID/g). This rise in radioactivity was significant (Fig 4D). The A549-pDNA tumors showed no difference in ^99m^TcO_4_^-^ uptake (% ID/g) compared to A549 control cells and remained constant over the two time points (Fig 4D).

### Quantification of hNIS expression in tumors

We corroborated hNIS expression by qRT-PCR of mRNA from excised orthotopic tumors from both the right and left lung (Fig 4E). Similar to our xenograft results, the A549-pDNA 1 tumor had low levels of hNIS expression, with an average of 0.45 for the relative quantification of A549-pDNA 1. Comparatively, the A549-LV tumor group exhibited robust relative mRNA levels, expressing 333-fold higher hNIS over the A549-pDNA 1.

### *Histopathological analysis* of primary tumors in the orthotopic tumor model

**Fig 5:**
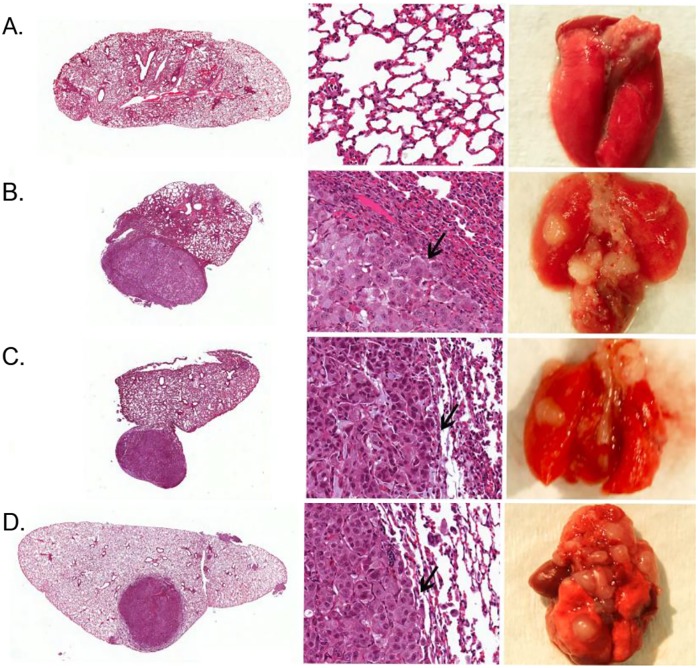
H&E staining of orthotopically-induced lung tumors in athymic Nude-Fox1nu mice. Tumors were induced by injecting A549-hNIS cells that were genetically modified with either plasmid or lentiviral vectors. Mice were sacrificed at 36 days post cancer cell inoculation. An H&E section of the lung is shown at 4x magnification (left), 60x magnification (middle; arrow marks tumor margin), or (right) gross specimen showing solitary nodules, from the induction of A) Saline control (no tumor). B) A549 lung adenocarcinoma control cell line; no genetic modification. C) A549-pDNA tumor 1. D) A549-pDNA tumor 2. E) A549-LV tumor (n = 3; representative images shown).

For the orthotopic tumor model, multiple tumor masses on the pleural surface and in the parenchyma of the lung were observed (Fig 5B–5E). Tumor nodules were found primarily in the left lung, where the A549 hNIS-modified cells were injected. Tumor masses were clearly distinguishable from adjacent lung tissue, and were morphologically similar to those seen at sites of transplantation. No signs of necrosis were visible in any of the tumors. Interestingly, intrapulmonary metastases were also observed in the right lung.

## Discussion

Animal models are critical for understanding the fates, effects, pharmacokinetics, and pharmacodynamic interactions of novel drugs. Although there is always uncertainty when extrapolating animal data to humans, preclinical data provides a baseline to assess drug efficacy and safety. Furthermore, animal models can recapitulate human disease and may provide insight into certain aspects of disease pathology, progression, and treatment. However, with respect to lung cancer, no reliable animal model currently exists [32].

At present, investigators must choose to either study drug effect or disease pathology and progression when using existing animal models. The s.c. induction of xenograft tumors is a common method of transplanting tumor cells or material in nude mice allowing researchers to directly measure the effect of drugs on tumor sizes and burden measured using a caliper [33]. However, researchers have questioned the accuracy of xenograft tumor data when applied to human drug trials [34,35]. Orthotopically-induced xenograft models are advantageous over s.c. xenograft models because the former are more physiologically relevant based on the tumors enhanced invasive and metastatic properties [34,36]. These properties are especially important in lung cancer where the cancer micro-environment plays an important role in therapeutic efficacy [37,38]. Thus, there is a need for the development of tissue-specific cancer models which can be monitored longitudinally using non-invasive imaging methods to adequately assess future cancer therapeutics. Our goal was to develop a longitudinal preclinical lung cancer imaging model using modified A549 human lung adenocarcinoma cells expressing the hNIS gene as a reporter gene. For this purpose, we constructed and characterized genetically modified lung cancer cell lines expressing the hNIS gene using two different means of gene transduction. We then developed s.c. and orthotopic xenograft mouse tumor models that could be longitudinally imaged by SPECT/CT to evaluate tumor growth kinetics non-invasively.

The hNIS gene has been utilized in thyroid imaging for decades and has many advantages as an imaging reporter gene [21]. Due to its long-established use in the clinic, it has several widely available radioisotopes and well-understood metabolism and clearance mechanisms. Previously, Chen et al. stably transfected MH3924A cells (rat hematoma) with a recombinant retroviral vector expressing functional NIS protein [39]. These *in vivo* studies yielded clear images of hNIS-expressing tumor xenografts using ^99m^TcO_4_^-^. Here, we translated Chen and colleagues’ work to an orthotopic lung cancer transplantation mouse model, similar to that developed by Kang et al, which simulated the clinical features of human lung cancer [39,32].

While there are other imaging modalities available for reporter gene imaging, SPECT/CT offers an attractive balance of sensitivity, resolution, tissue penetration, and affordability [40,41]. Modalities such as fluorescence and bioluminescence imaging are sensitive, however they are limited by tumor depth in the animal [5,42]. Magnetic resonance imaging (MRI) lacks sensitivity, and like positron emission tomography (PET), imaging can be expensive. Although PET is more sensitive than SPECT, the spatial resolution of SPECT is better than PET in small animal imaging [43]. SPECT/CT allows for the sensitive detection of reporters, in a relatively short time-frame with high resolutions and tumor penetration. Also, voxel number and intensity can be precisely quantified allowing for extremely accurate tumor burden quantification, which is important when using an orthotopically-induced tumor model, as tumors cannot be manually measured by calipers [44]. Here we validate the use of algorithmic modeling of SPECT scintillation, to precisely quantify tumor volume and burden by caliper measurement and H&E analysis by way of traditional animal sacrifice.

A secondary finding of this work was that significantly more hNIS expression and ^**99m**^TcO_4_^-^ uptake was achieved with A549-lentiviral vector-hNIS mediated tumors in both the s.c. and the orthotopic xenograft tumor model. The highest ^**99m**^TcO_4_^-^ uptake was observed in A549-LV cells, followed by A549-pDNA NIS F1 and F9 cells; these three cell lines were subsequently chosen for use in the *in vivo* tumor studies (Fig 1A). PCR results showed an average 17-fold increase of hNIS mRNA in A549-pDNA cell lines and a 2000-fold increase by the A549-LV cell line relative to A549 control cells (Fig 1B). These *in vitro* studies suggested stable incorporation of the hNIS reporter gene in A549 cells.

In our *in vivo* tumor studies, orthotopic implantation of tumor cells achieved a 100% tumor take rate confirming that the genetic modification of A549 cells did not cause the cells to lose its tumorigenic properties. Tumor growth was similar in all groups (Fig 4C). However, orthotopically-introduced A549-LV tumors expressed an average of 8-fold more radioactivity per gram of tumor (% ID/g) than A549 control tumors, whereas the A549-pDNA tumors showed no increase in radioactivity per gram tumor (% ID/g) compared to the A549 control tumors (Fig 4D). These findings are significant because they suggest that plasmid transfection with pDNA may not be the best option for robust reporter gene expression in animal models. However, it is important to note that A549-pDNA 1 tumor had detectable hNIS mRNA by qRT-PCR at both imaging time points in both the s.c. and orthotopic tumors (Figs 2F and 4E). Furthermore, the ^**99m**^TcO_4_^-^ uptake remained largely unchanged in both tumor models from the first time point to the second (Figs 2E and 4D). This suggests that hNIS incorporation was stable and functionality was retained over the course of the two time points analyzed. It may be that SPECT/CT is not sufficiently sensitive to detect less-robust gene reporters like the plasmid transfection described here. A significant increase in radioactivity per gram of tumor was seen in the A549-LV tumors between day 14 and 36 (Fig 4D). This may be due to the interpulmonary metastases seen in the A549-LV tumors (Fig 4B). These metastatic tumors contribute to radiotracer uptake (% injected dose of ^99m^TcO_4_^-^) but not toward the tumor mass, making the radioactivity (% injected dose of ^99m^TcO_4_^-^/per gram of tumor mass) appear higher.

Importantly, SPECT/CT was able to detect and quantify lentiviral-transduced tumors with sufficient sensitivity in both the s.c. and orthotopic xenograft tumor models. A549-LV tumors were imaged (Figs 2A–2C and 4A–4B) at two time points in both models, and SPECT/CT images were confirmed by actual tumor volume after animal sacrifice (Figs 3 and 5). Furthermore, A549-LV tumors had the highest radioactivity (% ID/g) of all tumors groups in both tumor models and this was maintained across experimental time points as tumors continued to grow (Figs 2E and 4D). This, in combination with the presence hNIS mRNA in A549-LV tumors (Figs 2F and 4E), suggest that we achieved stable hNIS integration into A549-LV tumors.

The limitations of this research are that SPECT/CT imaging and voxel quantification can be costly and are not widely available. Although the development and execution of the orthotopic tumor model containing genetically modified tumor cells is time-consuming, they are more clinically relevant to human lung cancer. Lastly, although we did not experience significant efflux of ^99m^TcO^4^- in either the s.c. xenograft or the orthotopically-induced tumor model, dynamic efflux of ^99m^TcO^4_^ in hepatoma and adenocarcinoma cells has been shown by other groups [18,45]. Stable incorporation of the hNIS gene, as shown here by the lentiviral transduction method, is the most crucial parameter for ^**99m**^TcO_4_^-^ uptake *in vivo*. Thus, we expect limited problems associated with ^99m^TcO4- efflux.

Future studies using the hNIS reporter gene should continue to focus on increasing the clinical relevance of these animal models to humans. Patient-derived xenograft (PDX) mouse models have the potential to increase our understanding of human tumor biology, as they utilize human primary tumor cells. However, PDX solid tumors, when grown s.c. in immunocompromised mice, do not metastasize [46]. Similar to the research described here, patient-derived orthotopic xenograft (PDOX) can lead to metastasis when implanted orthotopically in mouse models and thus are able to better recapitulate human tumors [47]. Since orthotopic mouse models necessitate non-invasive imaging to follow disease progression and response to therapy, future studies should establish genetically modified PDOX mouse models that could be imaged using small animal SPECT/CT and other imaging modalities. This is likely to improve the predictability responses from mouse models to those observed in patients as they better mimic the clinical patterns of tissue-specific human cancer and their metastasis.

This work describes the successful development of a clinically relevant lung cancer model which can be monitored longitudinally. Using the hNIS gene as an imaging reporter, we were able to monitor tumor growth and burden within the lungs at multiple time points within the same animal. This pre-clinical tumor model could significantly improve upon the existing lung cancer animal models by avoiding inter-animal variation and allowing long-term longitudinal monitoring of the same animal treated with novel anti-cancer drugs. Importantly, this methodology could be broadly translated to other tissue-specific cancers, and thus result in the use of the hNIS gene as an imaging reporter in other orthotopic cancer models.

## Supporting Information

S1 Fig Characterization of lentiviral vector-mediated hNIS expression in A549 lung cancer cells. Phospho Glycerat Kinase housekeeping gene (PGK) and Spleen focus forming strong viral (SFFV) promoter lentiviral-vector modified cells were characterized by surface hNIS expression percent positive and mean fluorescence intensity.
(TIF)
