# Supplementary figures and images for: Longitudinal Assessment of Lung Cancer Progression in Mice Using the Sodium Iodide Symporter Reporter Gene and SPECT/CT Imaging
Source: PLoS One. 2016 Dec 30;11(12):e0169107. doi: 10.1371/journal.pone.0169107 (PMC5201271; doi:10.1371/journal.pone.0169107)

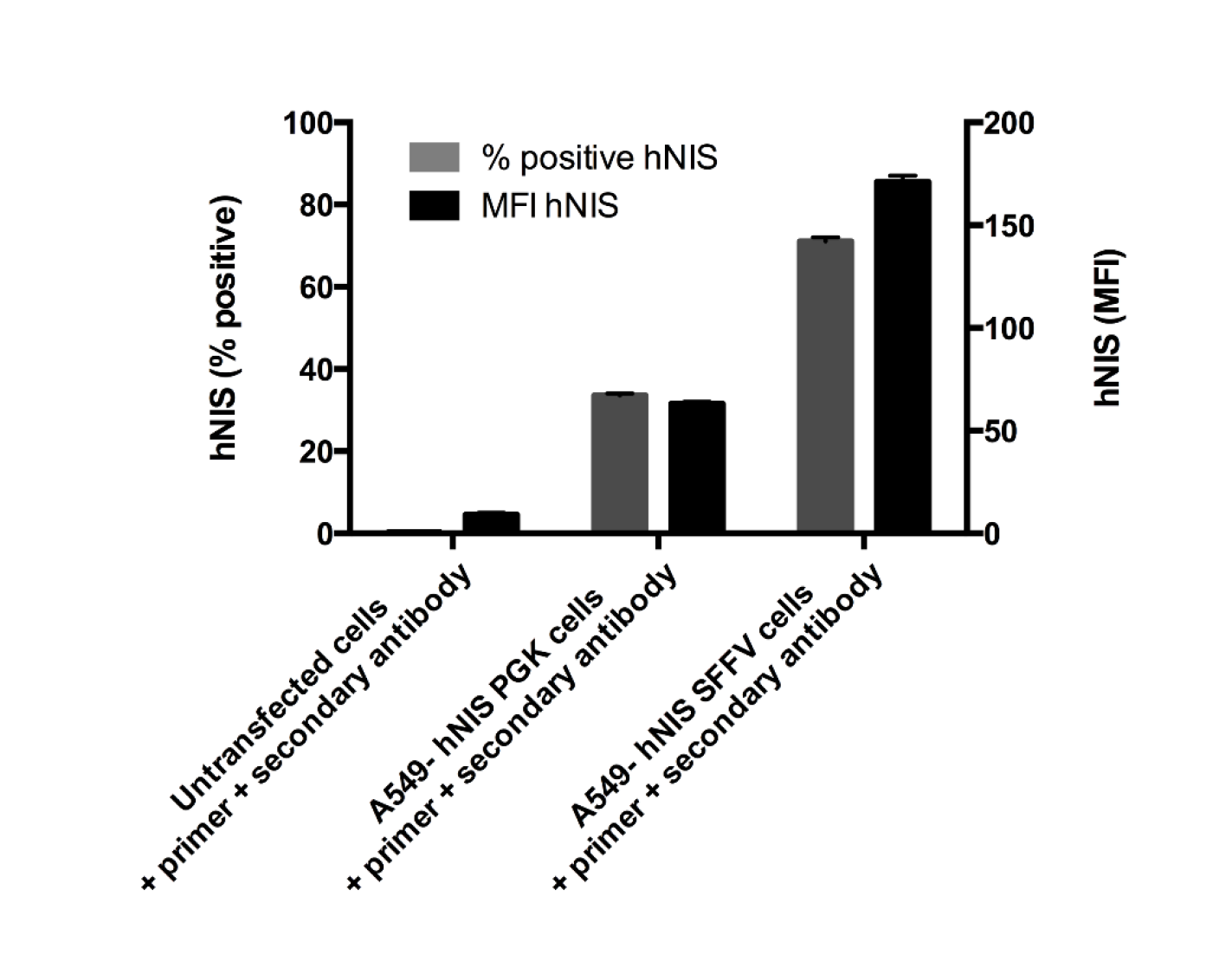

Supplement: S1 Fig — Phospho Glycerat Kinase housekeeping gene (PGK) and Spleen focus forming strong viral (SFFV) promoter lentiviral-vector modified cells were characterized by surface hNIS expression percent positive and mean fluorescence intensity. (TIF) [file pone.0169107.s001.tif]
